# Tuberculosis – eliminating the latent killer

**DOI:** 10.1016/j.lansea.2024.100385

**Published:** 2024-03-11

**Authors:** The Lancet Regional Health – Southeast Asia

Tuberculosis (TB) continues to kill people. In 2022, TB caused 1.3 million deaths globally and was behind only COVID-19 in causing the greatest number of deaths by a single infectious agent. TB is a disease of poverty, and its control is a major challenge for low-income and middle-income countries. The COVID-19 pandemic significantly disrupted TB services in the southeast Asia region. However, the region is slowly recovering. The numbers of reported deaths due to TB in the southeast Asia region in 2021 and 2022 were similar, and effective strategies need to be prioritised to further reduce the numbers drastically.

Delay in diagnosis continues to be a major challenge for TB elimination. A cohort study by Lestari and colleagues at a referral hospital in West Java province, Indonesia, revealed considerable delays in diagnosis and treatment of drug-resistant TB cases (rifampicin-resistant). Shockingly, around 50% of the cases had unsuccessful outcomes such as loss to follow-up, treatment failure, or death. The study also found that distance from the referral hospital and employment status were major factors that contributed to delay in diagnosis. Health authorities need to focus on disadvantaged populations—especially the socially disadvantaged—residing in urban slums and areas with difficult terrain.

People from lower socioeconomic backgrounds and daily wage earners are most likely to delay seeking health care and discontinuing treatment. Community health-care workers—interacting closely with vulnerable people in the community—have attempted to promote treatment adherence and access health care. But such close interactions are challenging, considering the risk of stigmatisation and issues of confidentiality. Nagarajan and colleagues interviewed people with drug-resistant tuberculosis and their caregivers in Bengaluru and Hyderabad, India, to understand how treatment adherence was achieved. Caregivers (i) ensured that people with tuberculosis took the tablets (by providing water on time), (ii) reminded people with tuberculosis the hassles of starting the treatment all over again, (iii) encouraged people with tuberculosis to think of children and dependents, and (iv) encouraged people with tuberculosis to consider pills as chocolates (‘positive labelling’). Consistent support from the caregivers and motivation from other people with tuberculosis was important for strict treatment adherence. This is critical for stopping continued transmission and antimicrobial resistance.

Incentives to people with tuberculosis can help. For example, India’s *Nikshay Poshan Yojana* scheme provides INR 500 (US$6) per month to people with tuberculosis visiting clinics for treatment. Shastri and colleagues in their Health Policy discussed how integration of health scheme *Ayushman Bharat-Pradhan Mantri Jan Arogya Yojana*-Arogya Karnataka (AB-PMJAY-ArK) with the National Tuberculosis Elimination Program (NTEP) helped TB patients in the state of Karnataka, India. Such integrations could be tested for feasibility in other settings. Furthermore, Karnataka's gatekeeping mechanisms made sure most people with tuberculosis were admitted to public hospitals rather than private hospitals, leading to reduced treatment costs.

Early screening is also essential for preventing TB transmission. Thailand is using portable AI-assisted X-ray units for chest X-rays, which help in quick screening before further diagnostic tests. Such portable X-ray units can be used for TB screening in remote and hilly areas too. Considering many countries invested in real-time PCR machines during the COVID-19 pandemic, these machines could be used for TB diagnosis, especially in cross-border regions of Nepal, India, and Myanmar. Hospitals can invest in upper-room germicidal ultraviolet light and mechanical ventilation systems in inpatient wards and natural ventilation in waiting areas for outpatients. Health authorities can involve standardised patients (healthy individuals trained to present with ‘fake’ TB symptoms) to regularly check the quality of TB diagnoses in hospitals. Although the BCG vaccine is well-known to prevent TB in children, it is not very effective in adults. Budi Gunadi Sadikin, Indonesia’s health minister, is pushing for the development of an all age-group TB vaccine by 2028 and aiming for TB elimination by 2030.

Globally, more than a billion people are estimated to have latent tuberculosis, with approximately 10% of cases reactivating into active infection. Nutrition plays an important role in positive treatment outcome and TB prevention. Countries, in the long run, should aim to reduce indoor air pollution and improve economic conditions, which would be beneficial in decreasing TB incidence. We need stronger polices on prevention, early diagnosis, treatment cascades, and treatment adherence, as well as a strong work policy for people with tuberculosis. Research on new vaccines and development of animal models that mimic human TB is the need of the hour. Striking the latent killer from all directions and in all possible ways would lead to elimination.

